# It’s easy to miss complicated hydatid cyst of lung

**DOI:** 10.4103/0970-2113.68328

**Published:** 2010

**Authors:** Debabani Biswas, Atin Dey, Saurabh Biswas, Mukul Chakraborty

**Affiliations:** *Department of Respiratory Medicine, Calcutta National Medical College, 24 Gorachand Road, Kolkata - 700 014, India*

**Keywords:** Air-bubble sign, pulmonary hydatid cyst, unintentional FNAC

## Abstract

A 60-year-old female presented with pneumonitis of right lower zone. CT scan revealed mass like lesion with multiple air pockets. FNAC and ultrasound confirmed the diagnosis as isolated active pulmonary hydatid cyst, which is not common finding in adult population.

## INTRODUCTION

Echinococcosis or hydatid disease, caused by larvae of the tapeworm *Echinococcus*, is frequently seen in an endemic country like India. Thevast majority of infestations in humans are caused by *E. granulosus.* The cyst may develop practically in any organ of the body. A single organ is involved in 85-90% cases, pulmonary involvement occurs in 10 - 30% of cases, being second only to the hepatic involvement.[1,2] However, in children the lungs may be the commonestsite of cyst formation.[3] Fikret Kanat *et al*.,^4^ have reviewed 134 patients operated retrospectively for pulmonary hydatid cysts over a period of 10 years and found that a concomitant hepatic cyst was present in 79% cases of adult as compared to 33% in children and concluded that isolated pulmonary hydatid cyst is not common finding in adult population. A simple pulmonary hydatid cyst can arise anywhere in the lung, but is more common in the right side and the lower lobe.[5,6] A complicated hydatid cyst is defined as a cyst that had ruptured into the bronchus or pleural cavity, with or without infection.[7] An infected ruptured cyst presents as fever, purulent sputum, leukocytosis, and pericystic pneumonitis with or without lobar andsegmental pneumonia.[7]

We present a case of isolated pulmonary hydatid cyst in adult and the dilemma faced in the diagnosis. The relevant literature is reviewed.

## CASE REPORT

**Figure 1:**
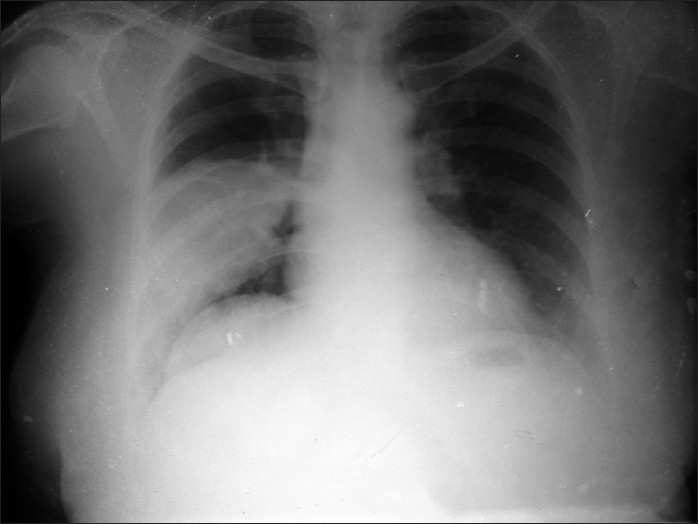
Homogenous opacity in right mid and lower zone

A 60-year-old Hindu female presented in our out-patient department with high rise of temperature with chills and rigor, and cough with minimal expectoration for two days. Clinically, the patient was febrile, alert conscious, tachycardic (120/min regular), tachypnic (28/min), blood pressure 100/60. Examination of the respiratory system revealed central mediastinum with impaired percussion note, decreased vesicular breath sounds and vocal resonance and a few crackles over right infra-axillary and infrascapular region. Review of the other systems revealed no abnormality. Routine blood examination revealed polymorphonuclear leukocytosis (Total count 18,000/cu.mm, Poly 80 lympho 16 Mono 2 Eosino2) and chest radiograph showed dense homogenous opacity with no air bronchogram in the right mid and lower zone [Figure 1]

**Figure 2:**
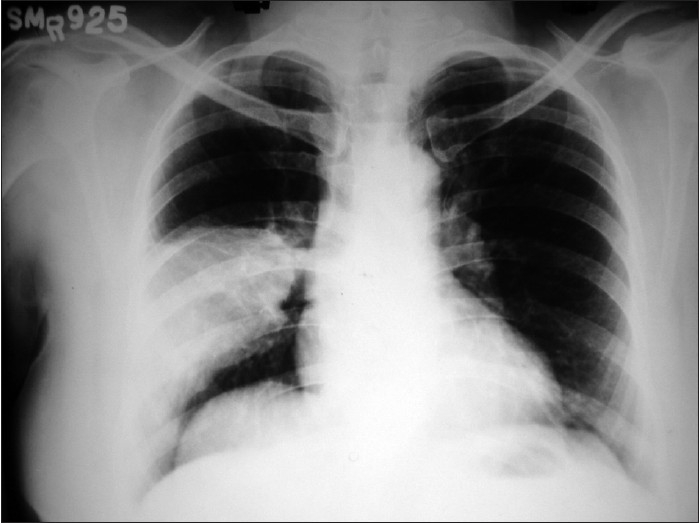
Homogenous opacity in same area after receiving antibiotic treatment for three weeks

Arterial blood gas report in room air revealed mild hypoxia (PaO_2_ 74 mm Hg). The patient was started on intravenous antibiotic and fluid. She became afebrile and hemodynamically stable within three days with improved peripheral leukocyte count. Intravenous antibiotic was replaced by oral antibiotics after ten days. Surprisingly, a repeat chest radiograph showed no significant improvement after three weeks of antibiotic treatment [Figure 2]. At this point the patient was in good general health with no specific complaint but impaired percussion note and diminished vesicular breath sound over the same area.

**Figure 3:**
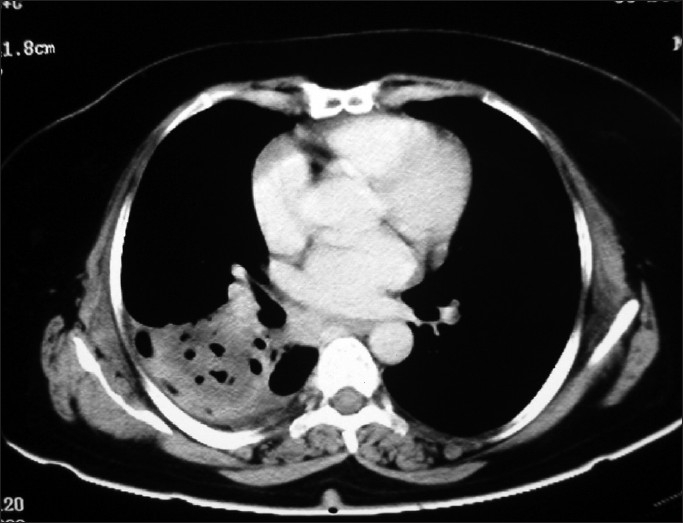
Mass with few air bubbles

**Figure 4:**
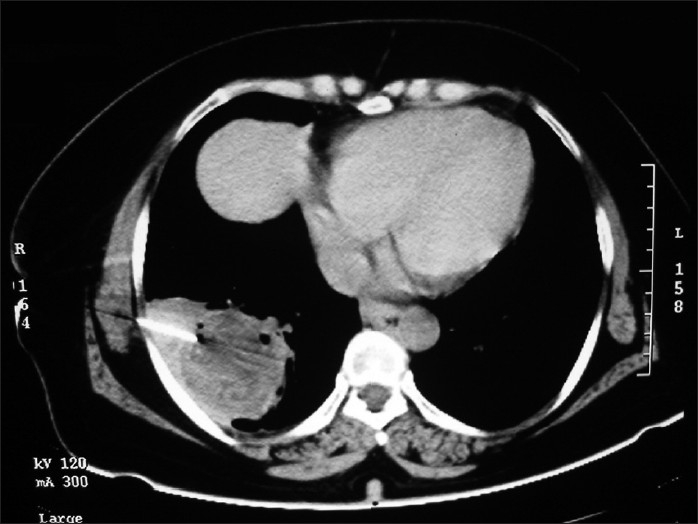
CT-guided FNAC

**Figure 5:**
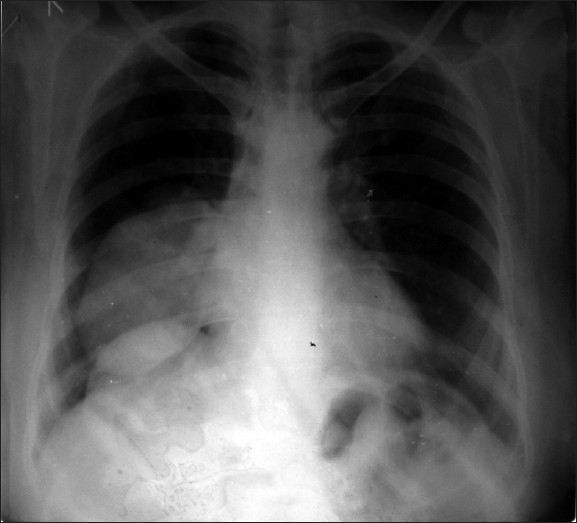
Well-circumscribed cystic lesion involving right mid and lower zone in year 2002

CT scan of thorax showed mostly homogenous mass in the right lower zone with few air bubbles inside and mild pleural effusion [Figure 3]. CT guided FNAC was done. But the report was inconclusive as it showed degenerative neutrophils along with a few lymphocytes and histiocytes on a necrotic background [Figure 4]. Fiberoptic bronchoscopy was done under local anesthesia, which excluded any intra-bronchial growth. While waiting for a repeat CT guided FNAC report of the mass we reviewed the detailed medical history including indoor and outdoor pet history. After repeated questioning she could remember that she suffered from ‘similar’ cough and cold about six years back and was treated with a course of antibiotic by a local doctor who said that there is a ‘patch’ in her lung [Figure 5]. She didn‘t follow-up with her doctor at the end of treatment as she was feeling ‘perfectly okay’. Also that she has had about 10 to 12 puppies and kitten as in-door pets since 15 years. ELISA for echinococcus antigen was advised and it came out significantly positive. The repeat FNAC report showed presence of fragments of lamellate membrane and scolises of echinococcus in the background of acute inflammatory cells. Ultrasonography and CT abdomen was done, which excluded the presence of cysts in diaphragm and other intra-abdominal organ.

## DISCUSSION

The purpose of reporting this case is to share an uncommon CT feature of pulmonary hydatidosis and emphasize the importance of eliciting past medical and pet history. Computerized search of MEDLINE, PubMed and Google database has been carried out using keywords pulmonary hydatidosis, CT appearances, fine needle aspiration cytology and serodiagnosis.

Most serodiagnostic techniques, to varying degree, have been shown to give false results, with considerable variation between laboratories. Overall, the diagnostic efficacy of ELISA is around 92.3%.[8] However, CT and radiography are the diagnostic methods of choice, considering high false positive results with serodiagnosis. Simple cysts are homogeneous, with a densityclose to that of water. Infection of the cyst may increase the attenuation values and present as a solid mass lesion causing diagnostic error.[9] CT scan with contrast may demonstratea thin enhancing rim if the cyst is intact. The ruptured membranes of hydatid cyst have been described as a number of different signs apart from the classically described “crescent”, “water-lily”, “double arch or cumbo’s” sign, for example “inverse crescent”, “signet ring”, high CT density like mass and thick walled cavity, “daughter cyst”, “ring within ring”, “mass within a cavity” or Monod’s sign, “serpent” or “snake” and “spin” or whirl" sign.[6,10] However, the demonstration of air-bubbles within the cyst together with ring enhancement, are strong indicators for infected hydatid cysts.[11]

Köktürk O *et al*.^12^ and G. Yuncu *et al*,[13] reviewed CT scan reports of a large number of patients with infected hydatid cyst that has been proven by other diagnostic methods including surgery. They concluded that the inclusion of air-bubble sign along with the classical CT features improves significantly the diagnostic accuracy of infected ruptured pulmonary hydatid cyst (Sensitivity ~ 85%, Specificity ~ 96%). Air-bubble sign has been included as a new CT sign indicating infected ruptured hydatid cyst.[11,12] The mechanism of air bubble production is the dissection of air between the pericyst and parasitic membrane due to rupture or erosion of a bronchiole.[14,15] Air bubble sign is best demonstrated in mediastinal window settings.

There is controversy regarding the role of fine-needle biopsy cytology in diagnosis of hydatid cyst considering chances of rupture of the cyst, anaphylaxis and dissemination. However, unintended fine needle aspiration of pulmonary hydatid cyst has been reported several occasions in order to obtain material for diagnosis of suspected mass lesion without any untoward complication and hence FNAC has been described as a safe diagnostic approach in the evaluation of suspected hydatid disease.[16–19]

Active cysts exhibit clear watery fluid containingscolices and show elevated pressure, whereas inactive cystsexhibit cloudy fluid without detectable scolices and do notshow elevated pressure. Even, the presence of acellular laminated membranes in fine-needle biopsy confirms the diagnosis of hydatid cyst.[17,18]

Our patient presented as pneumonitis of right lower zone. Her past medical history is suggestive of a similar episode six years back. The age of the patient, persistence of lesion after antibiotic course, and the presence of a mass like lesion with high attenuation in CT scan led to the suspicion of intrabronchial growth. However, past medical records, long duration of illness, good general health, and paucity of signs and symptoms indicate benign nature of the disease. Finally, diagnosis was confirmed by an unintentional fine needle aspiration of the mass lesion which demonstrated scolises indicating active hydatid cyst. The procedure was not followed by any complications.

The CT findings corroborated the diagnosis. Hence the presence of air-bubble sign within a mass like lesion in a patient at risk of hydatidosis (area of residence, pet history, and past medical history) should lead to the suspicion of infected hydatid cyst and FNAC has been proved to be a very safe diagnostic procedure in suspected hydatid cyst, contrary to the previous concept. The uniqueness lies in the fact that this is a case of isolated pulmonary hydatid cyst which is not common in adult population.
